# Shared and Unique Features of Human Interferon-Beta and Interferon-Alpha Subtypes

**DOI:** 10.3389/fimmu.2020.605673

**Published:** 2021-01-19

**Authors:** Megen C. Wittling, Shannon R. Cahalan, Eric A. Levenson, Ronald L. Rabin

**Affiliations:** Division of Bacterial, Parasitic, and Allergenic Products, Office of Vaccines Research and Review, Center for Biologics Evaluation and Research, US Food and Drug Administration, Silver Spring, MD, United States

**Keywords:** type I interferon, interferon-beta, interferon-alpha, interferon-omega, human, primate

## Abstract

Type I interferons (IFN-I) were first discovered as an antiviral factor by Isaacs and Lindenmann in 1957, but they are now known to also modulate innate and adaptive immunity and suppress proliferation of cancer cells. While much has been revealed about IFN-I, it remains a mystery as to why there are 16 different IFN-I gene products, including IFNβ, IFNω, and 12 subtypes of IFNα. Here, we discuss shared and unique aspects of these IFN-I in the context of their evolution, expression patterns, and signaling through their shared heterodimeric receptor. We propose that rather than investigating responses to individual IFN-I, these contexts can serve as an alternative approach toward investigating roles for IFNα subtypes. Finally, we review uses of IFNα and IFNβ as therapeutic agents to suppress chronic viral infections or to treat multiple sclerosis.

## Introduction

**Figure 1:**
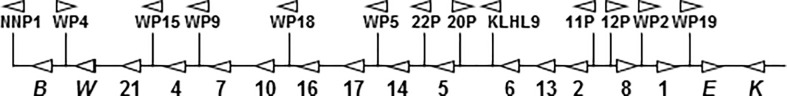
Gene map of the human IFN-I gene cluster. Above the line are pseudogenes for IFNν (NNP), IFNα subtypes, IFNω, and for the functional KLHL9 gene. On the line are the 17 functional type I IFN genes. Genes for IFNα subtypes are labeled only by number.

Type I interferons (IFN-I) are monomeric cytokines that are best known for their antiviral activity but that also suppress proliferation of cancer cells and modulate innate and adaptive immune responses. IFN-I were first discovered as an antiviral factor by Isaacs and Lindenmann in 1957 and were subsequently revealed to include IFNβ and multiple subtypes of IFNα (1, 2). We now know that human type I IFNs comprise a family of 17 functional genes and 9 pseudogenes clustered on chromosome 9 (3) that encode 16 proteins: IFNβ, ϵ, -κ, -ω, and 12 subtypes of IFNα (**Figure 1**). Since protein sequences for mature IFNα1 and IFNα13 are identical, we will collectively refer to them as IFNα1.

IFNβ may be considered the “primary” IFN-I because it is expressed by all nucleated cells and may be expressed in isolation of all other IFN-I (except IFNα1, discussed below). Two IFN-I genes are selectively expressed in specific organs or by specific cell types: IFNϵ is hormonally regulated and primarily expressed in the female genital tract (4) and has recently been reviewed elsewhere. IFNκ is primarily expressed by keratinocytes (5) where it has recently been shown to have a role in protection against cutaneous herpes simplex virus (6), papilloma virus (7), and cutaneous lupus erythematosus (8). Like IFNϵ, IFNκ is constitutively expressed (9). By contrast, IFNκ expression is activated and suppressed by TGFβ and ERK1/2 kinases, respectively (7, 10).

While IFNω is the least studied IFN-I in human biology, feline IFNω is well characterized and licensed as a veterinary antiviral therapeutic. In felines, IFNω is leukocyte specific (11, 12). While little is known about IFNω expression patterns, the presence of neutralizing autoantibodies is indirect proof that it is expressed and suggest a role in human disease. For example, in 2006, Meager et al. reported that 100% of *AIRE*-deficient patients with the autoimmune polyendocrinopathy syndrome have high titers of neutralizing autoantibodies against both IFNω and IFNα (13). More recently, Bastard et al. reported that ~1% of patients with severe Covid19 has selective neutralizing auto-antibodies against IFNω (14), suggesting that the importance of this type I IFN is in viral infections is underappreciated.

Mature IFNβ and eleven of the 12 IFNα subtypes are 166 a.a. in length (IFNα2 is 165 a.a. due to deletion of D44) with a MW of ~20 kD. IFNϵ and IFNω are 187 a.a. and 174 a.a., respectively, both due to an elongated carboxy-terminal, while IFNκ is 179 a.a. due to an insertion following residue 116. As shown in **Supplemental Figure 1** (15), IFNβ and IFNω share 31%–38% and 55%–60% identity with all IFNα subtypes, respectively, whereas identity among the IFNα subtypes ranges from 76%–96%. IFNβ, IFNω, and two IFNα subtypes are glycosylated; IFNβ at N80 (16), IFNω at N78 (17), IFNα2 at T108 (18), and IFNα14 at N72 (19).

Despite sharing only ~30% identity across all IFN-I, the three-dimensional structures are remarkably similar (20, 21). The salient structural features of all IFN-I, which are reviewed in detail by Walter et al. in this series include: 1) cylindrical proteins that consist of five 11-24 residue α-helices (labeled A–E), each parallel to the long axis of the cylinder; 2) Loops that connect the helices, of which the AB loop is relatively long and includes three short 3_10_ helices (22, 23); 3) conserved bonding including disulfide bridges (one in IFNβ, two each in IFNω and all IFN subtypes) and a network of hydrogen bonds to form and stabilize the tertiary structure; 4) IFNAR2 binding residues in Helix A, the AB loop and Helix E, and IFNAR1 binding sites spaced among helices B–D and the CD loop (21).

**Figure 2:**
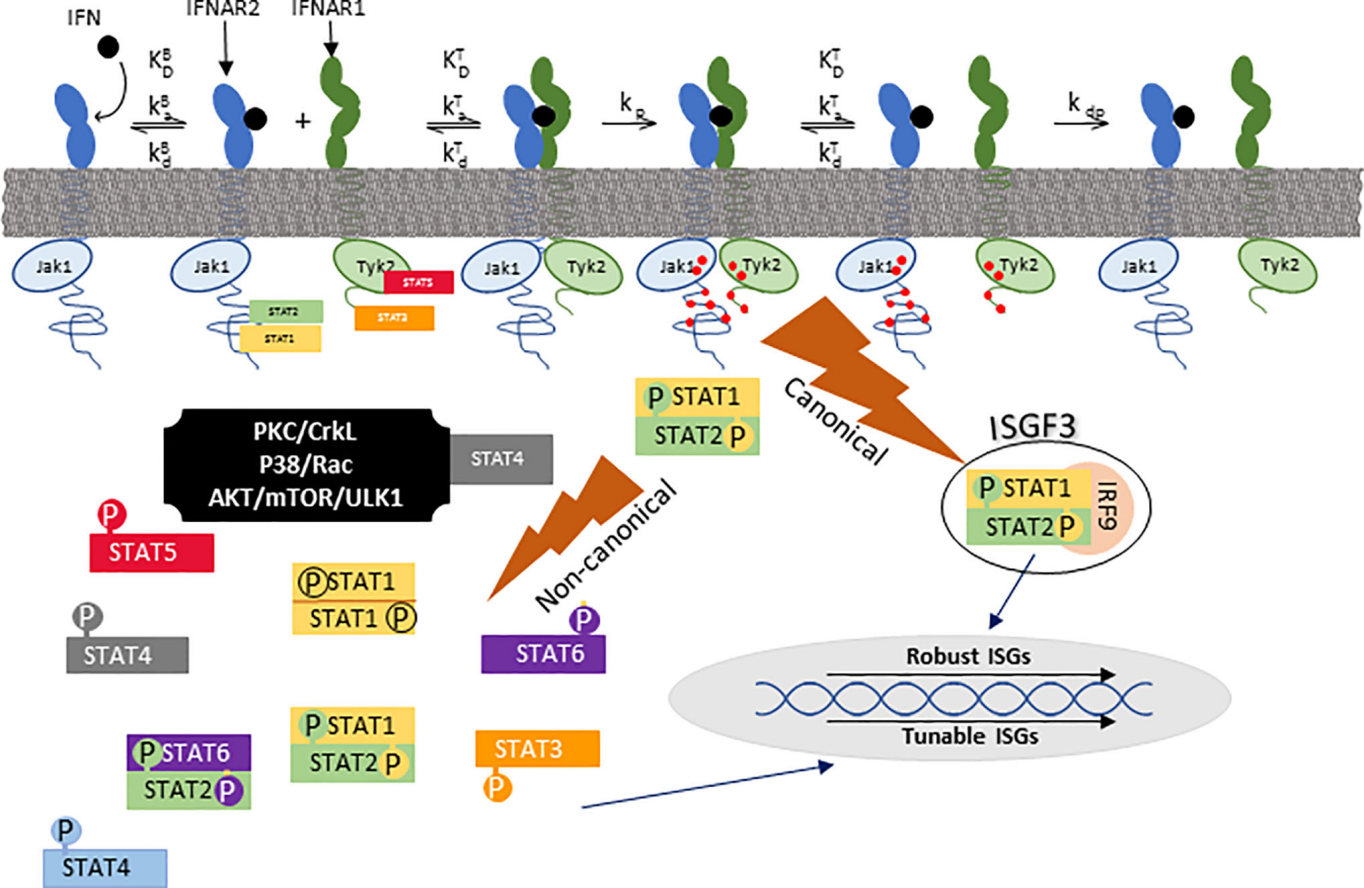
Canonical and noncanonical IFN signaling. IFN first binds to IFNAR2 after which the IFN/IFNAR2 binary complex recruits IFNAR1 to form a functional ternary signaling complex (IFN/IFNAR1/IFNAR2). Following that, Jak1 and Tyk2 kinases, which are pre-associated with IFNAR2 and IFNAR1 respectively, phosphorylate each other and tyrosine residues on each receptor (red dots) upon which STAT (signal transducers and activators of transcription) family members dock. Canonical signaling consists of a trimer of pSTAT1, pSTAT2, and IRF9 which is referred to as ISGF3 (interferon-stimulated gene factor 3). ISGF3 translocates to the nucleus to bind ISRE (interferon-stimulated response elements) to stimulate transcription of robust ISGs. There are many non-canonical signaling pathways, one of which is formation of phosphorylated STAT1 homodimers that bind to GAS (gamma activation site) promoter elements. k_a_ and k_d_ are association and disassociation rates, respectively. K_D_ is the equilibrium disassociation constant (k_d_/k_a_). k_p_ and k_dp_ are rates of phosphorylation and dephosphorylation, respectively. K^B^ and K^T^ refer to binary (IFN/IFNAR2) and ternary (IFN/IFNAR2/IFNAR1) complexes, respectively. This figure was adapted from **Figure 1** of (24).

All IFN-I signal through a heterodimeric receptor that is comprised of two subunits, IFNAR1 and IFNAR2. In the classical model of IFN signaling, IFN first binds IFNAR2 forming a high-affinity binary complex which then recruits IFNAR1 to form a functional ternary structure that triggers phosphorylation of Jak1 and Tyk2-initiating “canonical” signaling (24). In canonical IFN-I signaling (**Figure 2**), activation of Jak1 and Tyk2 is followed by phosphorylation of STAT1 and STAT2, which trimerize with IRF9 to form the transcription factor interferon-stimulated growth factor-3 (ISGF3) (25). Once assembled, ISGF3 translocates to the nucleus and binds to interferon stimulated response elements (ISRE) to promote transcription of interferon stimulated genes (ISGs). Through this canonical pathway, many genes are highly susceptible to shifts in expression with small amounts of IFN-I, thus earning the label of “robust” ISGs (26). Robust ISGs include most antiviral effectors from which the name “interferon” was derived.

Non-canonical IFN-I signaling includes cell-specific pathways such as those mediated by STAT1 homodimerization, other STAT family members, and MAP- or PI_3_-kinases (**Figure 2**). To better characterize these pathways, Urin and colleagues used HeLa cell signaling-component deletion mutants to show that except for the formation of STAT1 homodimers or STAT2/IRF9 heterodimers, non-canonical signaling depends on phosphorylation of both STAT1 and STAT2 (27). For the most part, non-canonical signaling induces “tunable” ISGs (26), which exhibit gradual rather than steep dose-response curves, and higher IFN concentrations for peak expression (26). Non-canonical pathways such as suppression of cell proliferation best correlates with the stability of the IFN/IFNAR1/IFNAR2 ternary complex [defined as (IFN-I K_D_ IFNAR1* IFN-I K_D_ IFNAR2)] (24). Non-canonical signaling also mediates expression of chemokines and cytokines that modulate innate or adaptive immunity, transcription factors that modulate cell phenotype, and some antiviral responses. As examples, APOBEC3, a cytidine deaminase that blocks HIV replication in macrophages, and IRF1, a transcription factor that mediates IFN-dependent and -independent viral immunity (28–31), share characteristics of tunable ISGs. While IFNAR2-independent signaling has been reported in mice (32), there are no data to controvert the current model that both IFNAR1 and IFNAR2 are necessary for signaling in humans.

Why there are so many IFN-I genes, and specifically so many IFNα subtypes, remains a mystery. As would be predicted by their common use of a shared receptor, evidence to date points to quantitative rather than qualitative differences among the IFN-I. In other words, differences in gene expression, antiviral, or antiproliferative activity at subsaturation are equalized by dose adjustments or in the extreme, by receptor saturation. Thus, while their evolutionary history and expression patterns suggest that at least some IFN-I serve specific functions, very few have been defined. Here, we focus on differences among IFNβ and the IFNα subtypes to propose a model by which patterns of expression mirror their evolutionary history, and thus provide an alternative approach toward deciphering their roles in human biology.

## Evolution of Type I Interferons

**Figure 3:**
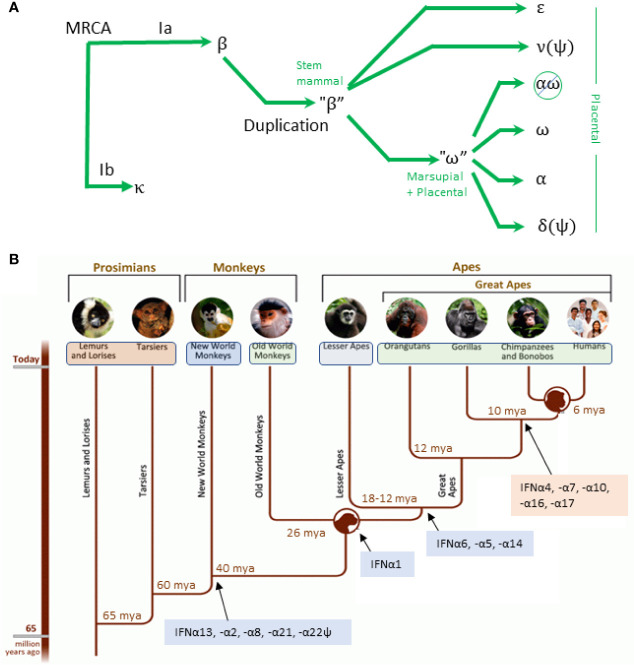
Evolution of IFN-I. **(A)** Simplified evolution of type IFN-I in mammals adapted from Krause and Petska. The most recent common ancestor (MRCA) gave rise to IFNκ and a progenitor for IFNβ. A duplicate of the IFNβ progenitor gave rise to IFNϵ, IFNν (a pseudogene in mammals), and a progenitor for IFNω. The IFNω progenitor gave rise to the remaining subtypes. In simiiforms, IFNαω is deleted and IFNδ is a pseudogene. **(B)** Evolution of IFN subtypes from simians to homininae showing conserved (blue) and variant (orange) subtypes. Figure adapted from: http://humanorigins.si.edu/evidence/genetics.

Types I and III IFNs evolved from a common ancestor gene that shares the 5-exon/4-intron organizational structure of the IL-10 family of cytokines. The intronless IFN-I genes of all higher order primates evolved and diversified from those of cartilaginous and bony fish. As shown in **Figure 3A**, IFNκ was the first to evolve from the “most recent common ancestor” (MRCA), followed by IFNβ. Both were present ~200 million years ago (MYA) before eutherians and marsupials diverged. IFNε arose from IFNβ, which later duplicated to give rise to IFNω and the IFNα genes (15). Primate IFN-I are highly divergent from other mammals. For example, in bats and ungulates, IFNω emerged as a multigene subtype (33) while primates have one functional IFNω gene and multiple IFNα subtypes.

The first *IFNA* gene appeared 95–105 MYA, which through duplication and conversion gave rise to an expanded set of IFNα subtypes in a subset of placental mammals (15). *IFNA* gene duplication and conversion that occurred before speciation gave rise to a conserved cluster of IFNα subtypes that are dissimilar, but that are shared across species. Conversely, duplication after speciation gave rise to variant clusters that are highly similar within each species but are not shared across species. As shown in **Figure 3B**, the first IFNα subtypes that are present in humans and simiiforms—*IFNA13*, -*A2*, -*A8*, and -*A21*—were present before the divergence of new world and old world monkeys (NWM and OWM) 65-47 MYA. NWM have one gene each for *IFNA13* (syntenic with *IFNA13* in monkeys and apes), *IFNA2* and *IFNA21*, and two genes each that are similar to *IFNA8* and *IFNA5* in higher order primates. Subsequently, *IFNA13* duplicated to give rise to *IFNA1* (present in OWM and apes), and *IFNA5*, *IFNA6*, and *IFNA14* arose to complete the set of *IFNA* subtypes that are conserved during primate evolution (**Figure 3B**, blue background). The subset of human *IFNA* subtypes that are variant among primates (pink background) arose after orangutans and the other great apes diverged. It has been proposed that *IFNA4*, *IFNA10*, *IFNA17* are products of partial conversions from *IFNA14* or *IFNA21 (IFNA4*, *-A10*, and *-A17*) (15) and that *IFNA10* may have converted *IFNA7* or vice versa (34).

Based upon a detailed analysis of human polymorphisms in sub-Saharan African, Asian, and European populations, Manry et al. (35) found the fewest polymorphisms in *IFNA6*, *-A8*, *-A13*, and *-A14.* Exclusion of *IFNA1* from this group appeared to be based on the A137V substitution (residue 114 of the mature peptide), that is predicted to have no damaging effects, and in our experience, is not functionally different from A137 IFNα1 (36). Manry et al. concluded that these evolutionarily conserved subtypes have have undergone selection against nonsynonymous variants. Taken together, the conserved cluster may have evolved to counter pathogens common that threatened the MRCA to OWM and great apes, and there is a selective advantage for having two genes, *IFNA1* and *IFNA13*, that express IFNα1.

## Regulation of Type I Interferon Expression by IRF3 and IRF7

Comparing promoter regions and transcription factor usage provides insight toward specialized roles for the different IFN-I. The interferon regulatory factor (IRF) family members are the dominant transcription factors that regulate IFN-I expression. While IRF1, -2, -5, and -8 have been shown to regulate IFN-I expression, this review will focus on the two most important members, IRF3 and IRF7.

**Figure 4:**
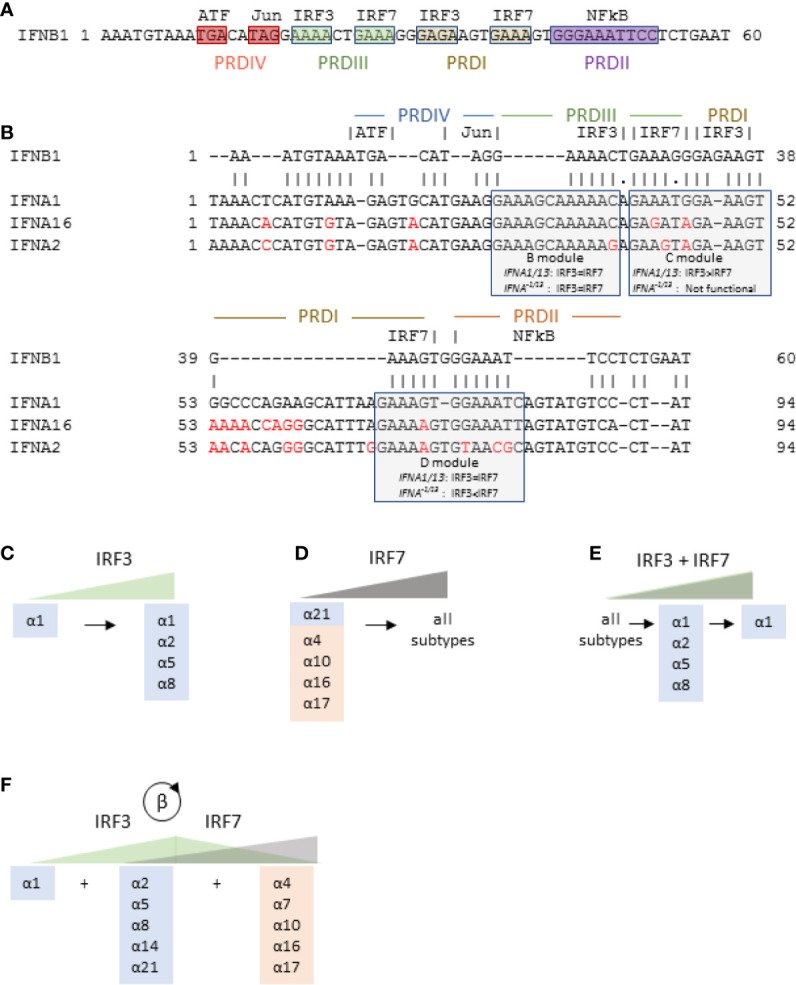
*IFNB1* and *IFNA* gene transcription is controlled by IRF3 and IRF7. **(A)** Promoter region of *IFNB1* gene showing the four promoter regulatory domains (PRD), all of which must be engaged for gene transcription. **(B)** Promoter regions of *IFNA1*, *IFNA16*, and *IFNA2* aligned with the promoter region of *IFNB1* showing the three IRF regulatory modules and their relative sensitivity to IRF3 and IRF7. Differences from *IFNA1* promoter are shown in red. The promoter region of *IFNA16* is representative of *IFNA21* and the variant subtypes (*IFNA17*, *IFNA16*, *IFNA10*, *IFNA7*, *and IFNA4)*. The *IFNA2* promoter region is representative of all IFNA*^−^* ^1/13^ conserved subtypes except *IFNA21.* **(C**–**E)** Model of differential regulation of human *IFNA* genes. Blue and orange shading show evolutionarily conserved and variant IFNα subtypes, respectively, *IFNA* genes expressed in response to increasing levels of activated IRF3 alone **(C)**, IRF7 alone **(D)**, or IRF3 and IRF7 together **(E)** as described by Genin et al. (38). **(F)** Proposed model of IFNα subtype expression in the context of initial activation of IRF3 followed by IRF7 expression (and subsequent activation) in response to a forward feedback loop initiated by IFNβ.

IFNβ is expressed after stimulation of pattern-recognition receptors (PRRs) such as RIG-I-like receptors (RLRs) and toll-like receptors (TLRs) by pathogen-specific molecular motifs referred to as pathogen associated molecular patterns (PAMPs) [reviewed in (37)]. Once activated, PRRs trigger signaling cascades that activate assembly of the “enhanceosome,” which consists of the transcription factors ATF-2/c-Jun, NFκB (p50/65 heterodimer) and two interferon response factor (IRF) dimers [**Figure 4** (39)] that bind to four promoter regulatory domains (PRDs). Based primarily on mouse models, it was initially thought that PRDs III and I required either IRF7 homodimers or IRF3/IRF7 heterodimers for a functional enhanceosome (40). In most cells, however, basal IRF7 expression is low while IRF3 is ubiquitously expressed. Thus, in most cells, viral PAMPs trigger activation of IRF3, which homodimerizes to complete the functional enhanceosome and initiate transcription of IFNβ. Subsequently, autocrine/paracrine IFNβ increases expression of IRF7 (a robust ISG) in infected and bystander cells—a well-documented critical step in a forward feedback loop for IFNβ to enhance its own expression (41).

The critical importance of IRF3 toward initiating IFN expression is emphasized by the number of pathogens with gene products that antagonize its activation (41) and by reports that cells from IRF3-deficient patients express little or no IFNβ (42, 43). The critical importance of the IRF7-mediated forward feedback loop is supported by an *in vitro* study in which the percentage of IFNβ-expressing cells after viral infection was dependent on cell density, and secretion of IFNβ (44), and reports that IRF7 deficient patients poorly express IFNβ (45, 46). By contrast, cells that constitutively express IRF7, as is the case for macrophages and plasmacytoid dendritic cells (pDC) (47) highly express IFN-I in response to synthetic ligands (imiquimod or CpG oligonucleotides) or pathogens such as influenza (48, 49). Taken together, the IFNβ-IRF7 forward feedback loop is a sentinel at the early stages of viral infection in local environments that enhances the antiviral state of common target cells for viral infection such as respiratory or gastrointestinal epithelium.

After the crystal structure of the *IFNB1* enhanceosome was published, Genin et al. described promoter regions of the *IFNA* genes (38) and modulated cellular expression of IRF3 and IRF7 to determine their effects on IFNα subtype expression. **Figure 4A** shows the promoter region of *IFNB1*, and **Figure 4B** shows the *IFNB1* promoter region aligned to representative *IFNA* subtypes up to −30 bp from the transcription start site. Overall, the *IFNA* promoter regions align well to that of *IFNB1* with 95% identity excluding several insertions and three short deletions. As shown in **Figure 4B**, the insertions into the *IFNA* promoters shift the IRF binding sites, referred to as modules B, C, and D, 5’ from the transcriptional start site such that the B module ends half-way through *IFNB1* PRDIII, the *IFNA* C module straddles *IFNB1* PRDIII and PRDI, and the *IFNA* D module straddles *IFNB1* PRDI and PRDII (to which NFκB binds in the *IFNB1* promoter). Among the three modules, only module B, which is equally responsive to IRF3 and IRF7, is essentially identical among all the subtypes. By contrast, module C, which preferentially binds to IRF3, is functional only in the *IFNA1 (and IFNA13)* promoter. Module D also differs between *IFNA1/13* and the other subtypes. For *IFNA1*, module D binds equally to IRF3 and IRF7, while for all the other *IFNA* subtypes, module D preferentially binds to IRF7. Binding of IRF3 to *IFNA1* promoter modules C and D explains why *IFNA1* and *IFNB1* can be co-expressed in the absence of any other *IFNA* subtypes (38, 49–51).

The promoter regions of the *IFNA* subtypes other than *IFNA1* (which we will refer to as *IFNA^−1/13^* or IFNα*^−^* ^1/13^ for the gene and protein, respectively) cluster into two groups. The first cluster consists of *IFNA4*, *-A7*, *-A10*, *-A16*, *-A17*, and -*A21*, (represented by *IFNA16* in **Figure 4B**). Note that this set includes all the evolutionarily variant *IFNA* subtypes (15) along with *IFNA21*, from which the variant subtypes may have arisen. The substitutions in the C modules of these subtypes renders them nonfunctional, and the 73G/A substitution in their D modules renders them more sensitive to IRF7. The B, C, and D modules are identical among the *IFNA* subtypes in this cluster.

The second cluster of *IFNA^−1/13^* subtypes is represented by *IFNA2* and includes *IFNA5*, *-A6*, *-A8*, and *-A14.* These are all evolutionarily conserved subtypes. The C module for this cluster is also non-functional, and their D modules include the 73G/A substitution that renders them more sensitive to IRF7. Unlike the cluster represented by *IFNA16*, however, there are substitutions in the B and D modules that may affect their relative sensitivity to IRF3 and IRF7 (52).

Based on analysis of the *IFNA* promoter regions and expression studies with EBV-transformed B cells, Genin et al. proposed a model for differential regulation of the *IFNA* genes by either activation of IRF3 or IRF7, or by co-activation of both IRF3 and IRF7 Genin, 2009 #71} (15, 52). In this model, low activation of IRF3 is sufficient to induce expression of IFNα1, while increased IRF3 activation may induce expression of IFNα2, -α5, and -α8 (**Figure 4C**). Similarly, increasing levels of IRF7 activation will first induce expression of IFNα21 and the evolutionarily variant subtypes followed by the remaining subtypes (**Figure 4D**). Co-activation of IRF3 and IRF7 at low levels induces expression of all subtypes, but coactivation increases, IRF3 inhibits IRF7 and thus limits the number of subtypes expressed (**Figure 4E**).

## Patterns of Human Type I Interferon Expression in Response to Synthetic Ligands and Viral Infection

To characterize expression patterns of IFNα subtypes in response to synthetic ligands or viral infection, transcripts are usually measured with RT-qPCR. **Table 1** summarizes human IFNβ and IFNα subtype expression patterns reported in the literature. As predicted by Genin et al., IFNα1 is co-expressed with IFNβ after activation of IRF3 with poly I:C. Additionally, when potently stimulated, pDC (which constitutively express IRF7) express all IFNα subtypes, while weaker stimulation of IRF7 with CpG B class oligodeoxynucleotides (ODN) induced expression of a set of IFNα subtypes that share the IRF7-sensitive promoter region exemplified by *IFNA16* (**Figure 4B**). By contrast, stimulation of cells that do not constitutively express IRF7 with viral RNA or the synthetic analog poly I:C primarily induces expression of a core set of conserved subtypes. **Table 1** also suggests the possibility that specific pathogens such as influenza virus, HIV, or hepatitis C may preferentially induce IFNα5.

**Table 1:** Reported expression patterns of human IFN-I.

Cell type	Stimulus		Conserved cluster						Variant cluster					CC	Reference
		β	α1	α8	α2	α6	α5	α14	α17	α16	α10	α7	α4	α21	
PBMC	poly I:C	X	X		X			X			X				(49)
	CpG B-D class	X	X	X	X			X		X	X	X		X	(53)
	Imiquimod	X	X		X			X							(53)
	Sendai Virus^a^	nd	X	X	X			X			X			X	(19)
	Hepatitis C virus		X				X								(54)
Mo	poly I:C	X	X					X							(49)
MDM	poly I:C	X						X			X				(49)
	CpG D class	X						X			X			
	M. tuberculosis	X	X												(50)
MDDC	poly I:C	X	X												(49)
	RSV	X	X	X	X			X						X	(55)
pDC	poly I:C, LPS	X	X				X	X							(49)
	Imiquimod	X	X	X	X	X	X	X	X	X	X	X	X	X
	CpG A, C, D	X	X	X	X	X	X	X	X	X	X	X	X	X
	CpG B class	nd							X			X	X	X	(48)
	IAV H1N1	nd	X	X	X	X	X	X	X	X	X	X	X	X	(48)
	HIV	nd	X	X	X	X	X	X	X	X	X	X	X	X	(56)
	HIV	nd	X	X	X		X	X							(57)
Calu3^b^	IAV H5N1 IAV pH1N1 SARS-CoV MERS-CoV	X					X								(58)
BEAS2B	RSV	X	X												(51)
Lung explants	IAV H3N2	nd	X	X	X			X	X		X				(59)
U937^c^	Sendai Virus (low MOI)	X	X	X	X	x	x	x	x	x	x	x	x	x	(60)
	Sendai Virus (high MOI)	X	X	X	X		X	X		X				X

a Expression patterns determined by mass spectrometry.

b Expression patterns determined by RNAseq, which may be insensitive to detecting highly identical transcripts.

c Lower case “x” refers to the IFNα subtypes that were not expressed after IFNAR2 blockade (see text).

PBMC, peripheral blood mononuclear cells; Mo, monocytes; MDM, monocyte derived macrophages; MDDC, monocyte derived dendritic cells; pDC, plasmacytoid dendritic cells; poly I:C, polyinosinic-polycytidylic acid; CpG, CpG oligodeoxynucleotides; HIV, human immunodeficiency virus; SARS-Cov, Severe adult respiratory syndrome coronavirus; MERS-CoV, Mideast respiratory syndrome coronavirus; MOI, multiplicity of infection. Blue and orange shading show evolutionarily conserved and variant IFNα subtypes, respectively.

Of particular interest is the report by Zaritsky et al., who infected the U937 histiocytic cell line with Sendai virus at low and high multiplicity of infections (MOI). While the U937 cells expressed all IFNα subtypes after infection at low MOI, expression was limited almost exclusively to the conserved set after infection at a high MOI. Furthermore, while IFNAR2 blockade (which repressed the IFNβ-IRF7 forward feedback loop) did not affect the expression pattern in the high MOI infection, it significantly repressed all subtypes except IFNα1, -α2, and -α8 after low MOI infection (60). Taken together, these studies support the model of Genin et al. in which activated IRF3 alone induces expression of conserved IFNα subtypes (**Figure 4C**), and IRF7 alone first induces IFNα21 and variant subtypes and subsequently induces expression of all subtypes (**Figure 4D**). In the context of the IFNβ-IRF7 forward feedback loop, however, **Table 1** suggests that conserved subtypes are first expressed, followed by variant subtypes (**Figure 4F**).

It is important to note that the evolutionarily conserved or variant IFNα subtype clusters are not expressed *en bloc*. One possible explanation is that unlike the variant subtypes, the B and D promoter modules vary by one or two bp, which may affect their relative sensitivity to IRF3 or IRF7 (52). Another factor is that IRF3 and IRF7 are not the only mediators of subtype expression. For example, a set of *IFNA* transcripts is regulated by a competing endogenous RNA (ceRNA) network. Kimura and colleagues first described stabilization of *IFNA1* transcripts by a natural antisense transcript (NAT) that spans the coding region and extends well beyond the 3’ poly-A UTR (61). They subsequently determined that the *IFNA1* NAT includes binding sites for microRNA-1270 (i.e., a microRNA response element) which otherwise represses *IFNA1* transcript levels. Additionally, NAT for *IFNA8*, -*A10*, -*A14*, and -*A17* (Kimura et al., personal communication) also sequester miRNA-1270 to enhance their transcript levels (62).

## IFNβ, the High-Affinity Sentinel

**Figure 5:**
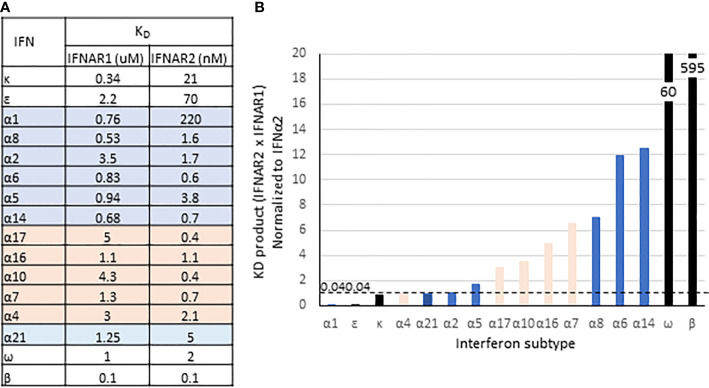
Binding affinities of IFN-I. **(A)** Equilibrium disassociation constants for the IFN-I. IFNα subtypes are from (63); IFNβ from (64), and IFNϵ, -κ, and -ω from (65). **(B)** Product of K_D_ for IFNAR1 and IFNAR2, normalized to IFNα2. Highlighting and bar colors indicate conserved (blue) and variant subtypes (orange).

In addition to its evolutionary emergence as the first non-tissue specific IFN-I and its high sensitivity to IRF3/IRF7, IFNβ also has exceptionally high affinities for IFNAR1 and IFNAR2 (K_D_ = 0.1 uM and 0.1 nM, respectively). As estimated by the product of IFNAR1 and IFNAR2 affinities (K_D_ IFNAR1 * K_D_ IFNAR2), the stability of the IFNβ/IFNAR1/IFNAR2 ternary complex is 10-fold higher than for IFNω and at least 50-fold higher than the highest affinity IFNα subtypes, IFNα14 and IFNα6 (**Figure 5**). As reviewed elsewhere in this series, a consequence of its high affinity is more effective internalization of ternary receptor complexes (66) into early endosomes where signaling may be amplified and prolonged, or more rapidly terminated due to shuttling of IFNAR1 to proteasomes for degradation (67).

A second consequence of the high affinity that IFNβ has for the receptor is that unlike the other IFN-Is, signaling is unaffected by ubiquitin-specific protease-18 (USP18). USP18 is a deubiquitinating enzyme that deconjugates the ubiquitin-like interferon-stimulated gene-15 (ISG15) from its target proteins (68). Conversely, ISG15 prevents ubiquitination and proteolytic degradation of USP18, thus stabilizing its expression (69). Unrelated to its enzymatic function, USP18 is shuttled by STAT2 to IFNAR2, which sterically blocks binding of Jak2 to interfere with recruitment of IFNAR1 to assemble a stable ternary complex (68, 70, 71). Since USP18 is an ISG (72), this inhibitory function is considered a negative feedback regulator of IFN signaling. Due to its very high affinity for IFNAR1, however, IFNβ can override USP18 and recruit IFNAR1 to form a ternary complex to initiate signaling (70). Thus, the negative feedback regulation by USP18 is selective and is presumed to affect all IFN-I other than IFNβ. To our knowledge, selective inhibition has been demonstrated by comparing IFNβ induced signaling with that of IFNα2, but not higher affinity IFNAR1 ligands such as IFNα6 or -α8, or those with higher IFNAR1 × IFNAR2 K_D_ products such as IFNω or IFNα14 (**Figure 5**). The critical importance of USP18-mediated inhibition of IFN signaling is exemplified by pseudo-TORCH syndrome, a severely incapacitating or fatal “interferonopathy” in patients deficient in ISG15, USP18, or with a mutation to the STAT2 binding site for USP18 (69, 73, 74).

Two additional qualities of IFNβ bear discussion. First, in addition to IFNκ (9), IFNβ also binds to highly sulfated proteoglycans (PG), proposed to be mediated through a heparin binding site in an arginine-rich region of IFNβ that spatially separates the binding sites for IFNAR1 and IFNAR2 (75). PG binding of IFNβ may sequester it to buffer IFN-I signaling, which can be reversed by desulfation or shedding the IFNβ-bound PG (75) which may result in a depot effect. Second, amino acid residues 25-27 uniquely contain the sequence motif NGR which binds CD13. Asparagine residues undergo spontaneous deamidation, which may be increased during oxidative conditions. Deaminated NGR gives rise to DGR, which binds to αVβ3 and possibly other integrins that similar to CD13, are expressed in blood vessels during angiogenesis (76) and by some cancer stem cells (77) and mediates tumor invasion (78). It is proposed that CD13 or αVβ3 in tumors or tumor vasculature may sequester IFNβ and thus limit its antiproliferative effects (79). In addition to these biologic effects, binding of IFNβ to abundant PG and integrins (in addition to its propensity to stick to plastic) may limit its detection in biological fluids or tissue culture supernatants.

## IFNα1, the Low-Affinity Subtype

**Figure 6:**
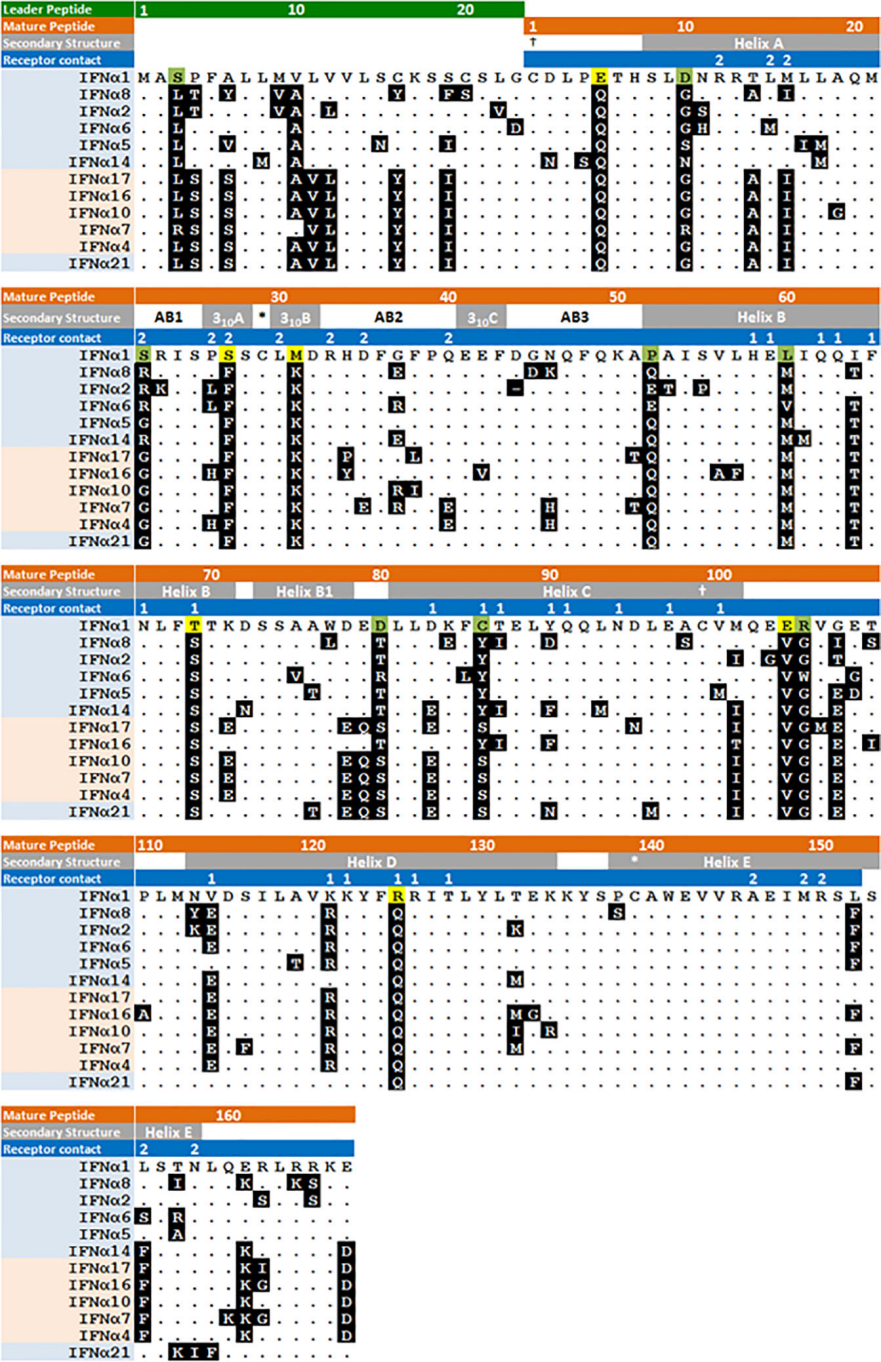
Amino acid sequence of human IFNα subtypes. IFNα subtypes are shown in order of arrangement on chromosome 9 with evolutionarily conserved and variant subtypes highlighted in blue and pink respectively. Secondary structure and IFNAR1/2 contact residues, labeled 1 and 2 respectively, are shown in the gray and blue highlighted text. Amino acids are shown with IFNα1 as the comparator, showing those that are unique to IFNα1 and otherwise identical among all the *IFN*α*^−1/13^* subtypes, or otherwise varies among the other *IFN*α*^1/13^* subtypes. * and † indicate cysteine disulfide bonds. Figure modified from (80).

As discussed above, IFNα1 stands apart from the IFNα*^−^* ^1/13^ subtypes for its responsiveness to IRF3, for having two genes (*IFNA1* and *IFNA13*) on chromosome 9, and for the low frequency of polymorphisms in either of those genes. Most remarkable, however, is the low affinity of IFNα1 for IFNAR2, at least 100-fold lower than most other IFNα subtypes while it binds with relatively high affinity to IFNAR1 (**Figure 5**). **Figure 6** shows the protein sequences of the IFNα subtypes aligned to IFNα1, with secondary structures and receptor contact points. Residues 20-35 cover most of the AB loop, including two 3_10_ helices. In this span, two substitutions in IFNα1 contribute to the low affinity of IFNα1 for IFNAR2: F27S, which decreases its affinity for IFNAR2 by 4-fold as the polar side chain of serine is predicted to disrupt the hydrophobic interaction otherwise stabilized by phenylalanine (80), and R22S, which together with S27 decreases affinity by ~14-fold (65). Although not a contact point, the substitution K31M in IFNα1 may also contribute to its decreased affinity for IFNAR2 by disrupting the second 3_10_ helix.

While the low affinity of IFNα1 for IFNAR2 suggests the possibility of a qualitative difference in signaling or functional outcome, the evidence to date only supports a quantitative difference. Reports of IFNAR2-independent signaling in mice (32) have not been replicated in human cells, for which it has been reported that both IFNAR1 and IFNAR2 are essential for signaling and gene expression (27). Additionally, while IFNα1 also has unique substitutions at contact points for IFNAR1 that may affect its conformation at the SD2-SD3 hinge that affect binding affinity (81), conformational changes do not necessarily indicate an effect in IFN signaling (25).

The substitutions that decrease the affinity of IFNα1 for IFNAR2 also decrease its affinity for B18R, a soluble receptor antagonist encoded by vaccinia virus. According to this model, secreted B18R (or other poxvirus orthologues) block high affinity IFN-I from binding their receptors, while leaving these low affinity IFNs relatively unaffected (65). Similarly, the organ-specific IFN-I, IFNκ, and IFNϵ also bind to IFNAR2 and B18R with low affinity. While IFNκ and IFNϵ may protect against poxviruses that infect local environments (skin and female reproductive tract), IFNα1 may defend against invasive strains such as variola. It is intriguing to speculate that the low frequency of polymorphisms in human *IFNA1* and *IFNA13* (35) is a consequence of a selective advantage toward surviving smallpox.

Among the IFNα*^−^* ^1/13^ subtypes, there are fewer substantial differences in their peptide sequences. **Figure 6** shows the shared residues that account for the high levels of identity among the evolutionarily conserved subtypes (**Supplementary Figure 1**) and differences in the unstructured C-terminal tail that contribute to higher antiviral and antiproliferative potencies of IFNα8 (82). Since the receptor contact points are conserved, variation in their binding affinities is apparently due to substitutions in adjacent residues.

## Therapeutic Uses of Type I Interferon

The antiviral and antiproliferative activities of interferons led to the development of their use as therapeutics. In 1986, IFNα2b (Intron A^®^, Merck Sharp & Dohme) was the first IFN-I approved for use in the United States (83). The current U.S. market for interferons, including IFNγ for chronic granulomatous disease and malignant osteopetrosis, has grown to $5B per year. **Table 2** shows the nine IFN-I licensed in the United States along with indications for use. As discussed elsewhere in this series of reviews (84), there are several ongoing clinical studies to test efficacy of IFN-I and IFN-III to treat Covid19.

**Table 2:** Licensed IFN-I in the United States.

Proprietary Name	Proper Name	Dosage Form	Dosage	Route	Indication	Expression System
Avonex	IFNβ-1a	30 µg/0.5 ml	30 µg per week	IM	Multiple sclerosis including relapsing-remitting and secondary active disease	CHO cells
Rebif	IFNβ-1a	8.8 µg/0.2 ml 22/44 µg/0.5 ml	22 or 44 µg 3 times per week	SC		CHO cells
Plegridy	IFNβ-1a	63/94/125 µg/0.5 ml	125 µg every 14 days	SC		CHO Cells
Betaseron	IFNβ-1b	0.3 mg	0.25 mg every other day	SC		*E. coli*
Extavia	IFNβ-1b	0.3 mg	0.25 mg every other day	SC		*E. coli*
Pegasys	Peg IFNα2a	180 µg	Adult: 180 ug per week Pediatric: 180 ug/1.73 m^2^	SC	Chronic Hepatitis C, Chronic Hepatitis B	*E. coli*
Pegintron	Peg IFNα2b	50/80/120/150 µg/0.5 ml	Adult: 1.5 ug/Kg/week Pediatric: 60 ug/m^2^/week	SC	Chronic Hepatitis C in patients with compensated liver disease	*E. coli*
Intron A	IFNα2b	10/18/25 MIU	Diagnosis Dependent	IV, IM, SC, IL	Hairy Cell Leukemia, Malignant Melanoma, Follicular Lymphoma, Condylomata Acuminata, AIDS-related Kaposi’s Sarcoma, Chronic Hepatitis C, Chronic Hepatitis B	*E. coli*
Sylatron	Peg IFNα2b	200/300/600 µg	6 ug/Kg/week for 8 weeks then 3 ug/Kg/week for up to 5 years	SC	Melanoma with metastasis to lymph nodes–to begin within 84 days of surgical resection	*E. coli*

Peg, polyethylene glycol; MIU, million international units; BSA; IM, intramuscular; IV, intravenous; IL, intralesional; SC, subcutaneous; CHO, Chinese hamster ovary cells.

IFNα2a or IFNα2b, which differ only at residue 23 (lysine or arginine, respectively), are prescribed for their antiviral or antiproliferative activity. These products are injectable preparations of either native or pegylated IFN proteins. Pegylation is modification of proteins with linear or branched polyethylene glycol to retards degradation and increase its serum half-life (85). While IFNα2 was used to treat chronic hepatitis C, it has been replaced with the highly specific inhibitors of HCV NS3/4A, NS5A, and NS5B proteins, which may be curative and are associated with fewer adverse events (86).

IFNβ was first approved for treatment of relapsing remitting multiple sclerosis in 1993 after showing an 18-34% reduction in relapse rate. The efficacy for IFNβ was considered to be due suppression of viral infections that are associated with relapses and to direct immunomodulatory effects that include reduction of pathogenic Th1 and Th17 CD4+ T cells, and to increases in IL-10 producing T_reg_ cells (87). All these may be mediated by increased expression of PD-L1 (CD274), an ISG that in mice is more responsive to IFNβ due to its high receptor affinity (88).

Therapeutic IFN-I has severe adverse events that are an obstacle to their use as therapeutics. The package inserts for pegylated IFNα includes black box warnings for the potential development of neuropsychiatric, autoimmune, ischemic, or infectious disorders. The package inserts also warn that treatment symptoms such as fever, fatigue, headache, myalgia, and nausea, which are usually associated with viral infections, are common side effects. More serious side effects can include cardiovascular and neurologic disorders, bone marrow, hepatic, and renal toxicity, and hypersensitivity reactions. Additionally, IFNβ for MS is associated with seizures, depression, suicide, and other psychiatric disorders. It is therefore not too surprising that as more selective therapeutic agents have been developed and licensed, use of IFN-I has become adjunctive rather than a primary treatment for chronic viral infections, cancer, or MS.

## Conclusions

As reviewed here, most if not all reported biological differences among IFN-I are quantitative rather than qualitative. While the antiviral subtype that most potently neutralizes infection *in vitro* may vary according to pathogen (57, 59, 89, 90), these differences may be overcome by increasing doses (57, 91). Similarly, differences in antiproliferative activity are largely dose dependent (92). While this may also be true for modulation of cytokine expression (36), immunosuppressive activity (i.e., induction of PD-L1) may be dependent on the exceptionally high affinity of IFNβ for IFNAR1/2.

As for the IFNα subtypes, other than escape from poxvirus soluble receptor antagonists (such as B18R by IFNα1), any suggestion of specialized roles is inferred from their evolutionary history or expression patterns. It is therefore possible that the primary role of IFNα is to prolong or amplify the effects of IFNβ and that multiple IFNα subtypes simply provide multiple layers of redundancy, albeit with a range of receptor affinities. However, it is also possible that unique functions for IFNα subtypes have not been revealed because the common experimental approach of comparing treatment with individual IFN-I does not reflect the biological context in which defined patterns of IFNα are co-expressed together and with with IFNβ. These patterns are likely most relevant at sub-saturating doses, which may more accurately reflect the environment of structural cells where organ specific immune responses are initiated (93).

## Author Contributions

MW, SC, EL, and RR performed the literature searches and contributed to draft versions of the manuscript. RR wrote and revised the final version of the manuscript. All authors contributed to the article and approved the submitted version.

## Funding

This study was supported by the Internal funds, US Food and Drug Administration. MW and SC were supported by an appointment to the Research Participation Program at the Center for Biologics Evaluation and Research administered by the Oak Ridge Institute for Science Education through an Interagency agreement between the U.S. Department of Energy and the U.S. Food and Drug Administration.

## Disclaimer

This article is an informal communication that represents the authors’ judgment. These comments do not bind or obligate FDA.

## Conflict of Interest

The authors declare that the research was conducted in the absence of any commercial or financial relationships that could be construed as a potential conflict of interest.
