# Supplementary figures and images for: Shared and Unique Features of Human Interferon-Beta and Interferon-Alpha Subtypes
Source: Front Immunol. 2021 Jan 19;11:605673. doi: 10.3389/fimmu.2020.605673 (PMC7850986; doi:10.3389/fimmu.2020.605673)

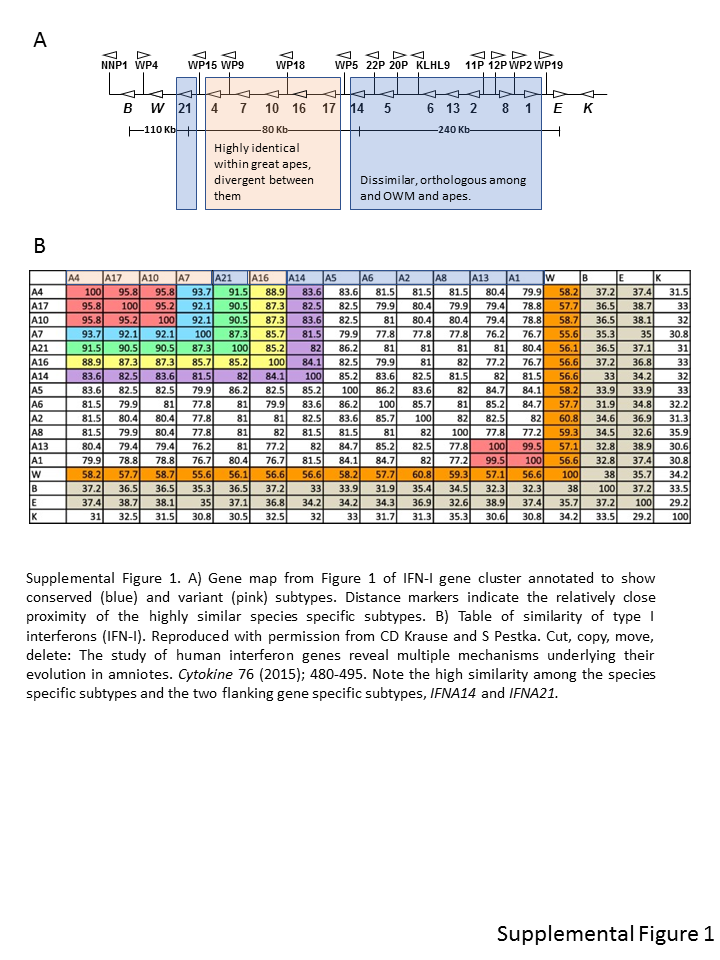

Supplement: Supplementary file 1 [file Image_1.tif]
